# One-Step Electrochemical Fabrication of Poly O-cresolphthalein Complexone and Electrochemically Reduced Graphene Oxide Modified Electrode for Detection of Nitrofurantoin

**DOI:** 10.3390/s26123682

**Published:** 2026-06-09

**Authors:** Ju Sung Kim, Da Eun Oh, Tae Hyun Kim

**Affiliations:** Department of Chemistry, Soonchunhyang University, Asan 31538, Republic of Korea; rlawntjd2009@sch.ac.kr (J.S.K.); dani0607@sch.ac.kr (D.E.O.)

**Keywords:** nitrofurantoin (NFT), electrochemically reduced graphene oxide (ERGO), O-cresolphthalein complexone (OC), antibiotic detection, electrochemical sensor

## Abstract

Nitrofurantoin (NFT) is a widely used antibiotic that requires sensitive and reliable detection due to its potential environmental and health impacts. In this study, a poly(O-cresolphthalein complexone)/electrochemically reduced graphene oxide (POC/ERGO) nanocomposite was electrochemically fabricated via one-step process and applied to a modified GCE for the electrochemical detection of nitrofurantoin. The sensing performance of the POC/ERGO-GCE was evaluated using CV and DPV. The developed sensor exhibited a wide linear detection range from 1 to 500 μM, and a low detection limit of 78.90 nM as determined by DPV. In addition, it demonstrated excellent anti-interference capability, good reproducibility and selectivity toward NFT, confirming the reliability of the proposed sensing platform. The enhanced performance is attributed to the increased electrochemically active surface area and improved electron transfer properties of the POC/ERGO-GCE. These results indicate that the proposed platform provides a reliable approach for the electrochemical detection of nitrofurantoin and offers a promising foundation for the development of antibiotic sensing systems.

## 1. Introduction

Nitrofurantoin (NFT), a representative nitrofuran antibiotic, has been extensively used in human and veterinary medicine because of its broad-spectrum antibacterial activity [1]. However, excessive use, clinical/veterinary administration, and improper disposal have contributed to its occurrence in biological, environmental, and food samples [2]. Consequently, NFT residues have been reported in environmental waters, food products, and biological matrices at trace concentration levels, raising concerns regarding antimicrobial resistance and potential toxicological effects associated with prolonged exposure to nitrofuran derivatives [2,3,4,5]. Therefore, the development of rapid, sensitive, and reliable analytical methods for NFT monitoring is important in environmental and biomedical analysis.

Various analytical methods have been developed for the determination of NFT, including liquid chromatography [6], spectrophotometry [7], fluorescence [8,9], and surface-enhanced Raman spectroscopy [10]. Although these methods provide reliable quantification, they generally require sophisticated instrumentation, complicated sample preparation procedures, and well-trained operators. In addition, the relatively long analysis time and high operational cost limit their applicability for rapid and on-site monitoring [11,12]. On the other hand, electrochemical sensing methods have attracted considerable attention due to their simple instrumentation, fast response, portability, and high sensitivity [11,13]. Moreover, NFT contains electroactive nitro functional groups that can undergo well-defined redox reactions, making it particularly suitable for electrochemical detection [2].

Recently, graphene-based nanomaterials have drawn significant attention as electrode-modifying materials for electrochemical sensors owing to their large surface area, and fast electron transfer characteristics [14]. In particular, electrochemically reduced graphene oxide (ERGO) can be directly formed on the electrode surface through a simple electrochemical reduction process, which is advantageous for facile and binder-free fabrication of sensing interfaces [15]. In addition, graphene-derived materials have already been employed in NFT electrochemical sensors, demonstrating that they are effective platforms for improving the electrochemical response toward NFT [16,17,18]. However, ERGO alone is often insufficient to provide a functional sensing interface because its contribution is mainly associated with facilitating interfacial charge transfer and increasing the electroactive surface area, while offering limited sites for favorable analyte interaction [15]. Therefore, further surface functionalization or hybridization with functional polymers is required to construct a more efficient sensing interface for NFT detection [15,19].

Functional polymer films have also been widely employed as sensing interfaces in electrochemical sensors due to their tunable chemical functionality and strong adsorption capability [20,21,22,23]. Electropolymerization provides a convenient strategy to form polymeric films directly on electrode surfaces, enabling controllable film thickness and stable surface modification [24,25]. The resulting polymer layers can introduce abundant functional groups, such as hydroxyl, carboxyl, and aromatic moieties, which facilitate molecular interactions with target analytes through hydrogen bonding, π-π interaction, and possible electrostatic attraction [26]. These properties make electropolymerized polymers attractive materials for improving adsorption-assisted electrochemical sensing [19,25]. However, many functional polymer films may exhibit limited intrinsic conductivity or hinder interfacial electron transfer when used alone, depending on their chemical structure and film thickness [27,28]. As a result, hybrid sensing interfaces that combine functional polymers with graphene-based materials have been actively explored to achieve both improved interfacial charge transfer and enhanced analyte accumulation [27,29]. Compared with conventional electrode assembly methods such as drop-casting and binder-assisted coating approaches, electropolymerization offers several practical advantages including conformal film formation, controllable film thickness, and direct deposition on conductive surfaces without additional binders [30,31]. Furthermore, electropolymerization enables localized and controllable assembly of functional materials at the electrode interface, which can be particularly advantageous for constructing hybrid sensing platforms where interfacial properties strongly influence sensing performance [30,31]. Among various electropolymerizable monomers, O-cresolphthalein complexone (OC) is particularly attractive because it is a triphenylmethane-based chelating dye containing iminodiacetic acid functionality [32]. OC has been extensively used as a metallochromic reagent for metal ion analysis, including calcium determination in complex samples and biological fluids [33]. The selection of OC as the electropolymerizable functional monomer was based on its molecular structure, which contains multiple aromatic moieties and oxygen containing functional groups, including hydroxyl- and carboxyl-related groups. These structural features may provide chemically favorable interfacial sites for NFT accumulation after electropolymerization. Specifically, the aromatic domains of POC may promote π-π interactions with the furan-containing conjugated structure of NFT, whereas hydroxyl-and carboxyl-related groups may contribute to hydrogen-bonding interactions with nitro, carbonyl, and hydrazone/imide-related moieties of NFT. In addition, oxygen-containing groups in the POC film may facilitate local enrichment of NFT through dipole-assisted and weak electrostatic interactions. Collectively, these molecular interactions are expected to enhance the interfacial preconcentration of NFT and facilitate its electrochemical reduction. Beyond its conventional analytical use, the electropolymerizable nature of OC enables the direct formation of a stable and functional polymer film on electrode surfaces [26]. Such a poly(O-cresolphthalein complexone) (POC) layer can also provide a stable sensing interface while maintaining favorable conditions for analyte accumulation and electrochemical signal transduction [34].

**Scheme 1:**
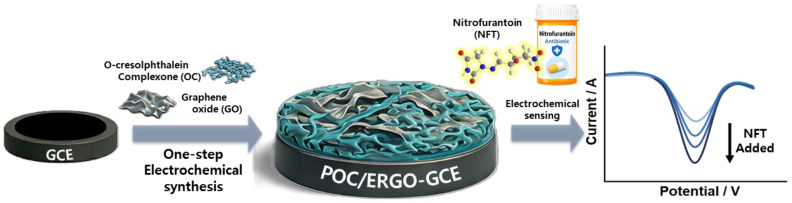
Schematic illustration of one-step electrochemical fabrication of POC/ERGO-GCE and its application for the electrochemical nitrofurantoin (NFT) detection.

Accordingly, integrating an electropolymerized functional film with an electrochemically reduced graphene oxide (ERGO)-based interface represents an effective strategy for constructing an efficient sensing platform for NFT detection. In the present system, POC can serve as a functional sensing layer formed directly on the electrode surface by electropolymerization, providing favorable interfacial site for interactive with and accumulation of NFT [26]. Meanwhile, ERGO can act as an electroactive support that facilitates interfacial charge transfer, increases the electroactive surface area, and provides a suitable substrate for the formation and integration of the POC film [35]. Such a hybrid configuration is particularly attractive for NFT sensing because the electrochemical response of NFT originates from the reduction of the nitro group, and both interfacial charge transfer and analyte accumulation at the electrode surface can strongly influence the sensing performance [5,16]. In addition, a one-step electrochemical process allows the simultaneous electro-polymerization of OC and reduction of GO, enabling the direct formation of an integrated POC/ERGO hybrid interface on the glassy carbon electrode (GCE) surface without additional binders or multistep modification procedures. Based on this strategy, a one-step electrochemical approach is proposed to construct a POC/ERGO-GCE for the sensitive detection of NFT, as illustrated in Scheme 1. Herein, we report a POC/ERGO-modified GCE prepared by one-step cyclic voltammetry in a mixed OC/GO solution for the electrochemical detection of NFT. The proposed sensing platform was designed to combine the functional contribution of POC with the interfacial charge transfer promoting characteristics of ERGO, thereby improving the electrochemical response toward NFT.

## 2. Materials and Methods

### 2.1. Materials and Instruments

Graphite powder, sulfuric acid (H_2_SO_4_), phosphate-buffered saline (PBS, pH 7.4, 10 mM), O-cresolphthalein complexone powder (OC, C_32_H_32_N_2_O_12_), nitrofurantoin (NFT, C_8_H_6_N_4_O_5_), and human serum were purchased from Sigma-Aldrich (St. Louis, MO, USA). Hydrogen peroxide (H_2_O_2_) was obtained from Daejung Chemicals & Metals Co., Ltd. (Siheung, Republic of Korea). Potassium permanganate (KMnO_4_), potassium ferricyanide (K_3_Fe(CN)_6_), potassium nitrate (KNO_3_), and dimethyl sulfoxide (DMSO, C_2_H_6_OS) were purchased from Duksan Reagents Co., Ltd. (Ansan, Republic of Korea). Sodium nitrate (NaNO_3_) was obtained from Samchun Chemicals (Seoul, Republic of Korea). Sodium hydroxide (NaOH) was purchased from SK Chemicals Co., Ltd. (Seongnam, Republic of Korea). All reagents were of analytical grade and used without further purification.

Electrochemical measurements were performed using a CHI 660D electrochemical workstation (CHInstruments, Inc., Austin, TX, USA, Z-202306208148 at the Research Support Center for Bio-Bigdata Analysis and Utilization of Biological Resources) and a CompactStat (Ivium Technologies, Eindhoven, The Netherlands). Ultrasonication was carried out using a Sonics Vibracell VC 505 (Sonics & Materials Inc., Newton, CT, USA) at 28% amplification with a pulse of 1 s on and 3 s off for 1 h. Raman spectra were recorded using an EnSpectr R532 Raman spectrometer (Enhanced Spectrometry, Inc., Austin, TX, USA, Z-202312061405 at the Research Support Center for Bio-Bigdata Analysis and Utilization of Biological Resources). Contact angle measurements were performed using a Phoenix 300 Touch automatic contact angle analyzer (Surface Electro Optics Co., Ltd., Suwon, Republic of Korea, Z-202312061406 at the Research Support Center for Bio-Bigdata Analysis and Utilization of Biological Resources).

Data analysis and curve fitting were performed using OriginPro 2018 (OriginLab Corporation, Northampton, MA, USA). Electrochemical impedance spectroscopy (EIS) data were analyzed using ZSimpWin software 3.60 (EChem Software, Ann Arbor, MI, USA). Electrochemical measurements were controlled and analyzed using CHI software v12.04 (CHInstruments, Inc., Austin, TX, USA) and IviumSoft 4.1239 (Ivium Technologies, Eindhoven, The Netherlands).

### 2.2. Electrochemical Measurements

Electrochemical measurements were carried out at ambient temperature using a conventional three-electrode setup. A glassy carbon electrode (GCE), ERGO-GCE, and POC/ERGO-GCE were used as the working electrode, while a Pt wire and an Ag/AgCl electrode served as the counter electrode and reference electrode.

ERGO modified GCE was prepared by cyclic voltammetry (CV), sweeping potential from −1.5 to 0.8 V (vs. Ag/AgCl) in a 10 mM PBS solution containing 0.3 mg/mL GO solution. The POC/ERGO-GCE was fabricated through a one-step synthesis in 10 mM PBS containing 0.3 mg/mL GO and 0.3 mM OC. For this process, CV was performed on the GCE at a scan rate of 50 mV/s for 5 cycles between −1.5 to 2.24 V (vs. Ag/AgCl), enabling the simultaneous reduction of GO and electropolymerization of OC [26,36].

For the electrochemical characterization, CV measurements were recorded by sweeping from −0.2 to 0.6 V (vs. Ag/AgCl) in 0.1 M KNO_3_ supporting electrolyte containing 10 mM Fe(CN)_6_^3−^. Electrochemical impedance spectroscopy (EIS) measurements were performed at the initial potential, calculated as ((E_pc_ + E_pa_)/2) from CV, under the following conditions: frequency range of 10^6^ to 10^−1^ Hz and an amplitude of 0.01 V.

Electrochemical detection of NFT was evaluated by CV and differential pulse voltammetry (DPV). CV measurement was sweeping a potential from −0.8 to −0.1 V (vs. Ag/AgCl). DPV measurements were conducted from 0.0 to −0.8 V (vs. Ag/AgCl) with the following settings: pulse amplitude of 70 mV, potential increment of 5 mV, pulse width of 50 ms, sample width of 16.7 ms, and pulse period of 0.5 s.

### 2.3. Preparation of O-Cresolphthalein Complexone (OC) Solution

To prepare the O-cresolphthalein complexone (OC) stock solution, exactly 19 mg of OC was weighed. To ensure complete dissolution, the solid powder was initially dissolved in 3 mL of 0.1 M NaOH aqueous solution. The resulting solution was then diluted with 7 mL of 10 mM phosphate-buffered saline (PBS, pH 7.4). The mixture was thoroughly homogenized to obtain a final volume of 10 mL with an OC concentration of approximately 3 mM.

### 2.4. Synthesis of POC/ERGO-GCE

Graphene oxide (GO) was synthesized using a procedure similar to the modified Hummers method [37,38]. Briefly, the graphite powder was mixed with NaNO_3_ at a ratio of 2:1 (*w*/*w*). A certain amount of H_2_SO_4_ (99.9 wt%) was added to the graphite-NaNO_3_ mixture, and the resulting suspension was continuously stirred at room temperature for 1 h to obtain a homogeneous suspension. Thereafter, KMnO_4_ was gradually added in amount at least three times that of the graphite-NaNO_3_ mixture with stirring at 40 °C for 12 h. The mixture was diluted with 50 mL of DI water to terminate the reaction. The resulting GO solution was purified by repeated centrifugation at 13,000 rpm. Finally, the purified GO dispersion was diluted to a concentration of 1.0 mg/mL using DI water. Homogeneous exfoliation was ensured by sonicating the 1.0 mg/mL GO dispersion in DI water for 1 h, which yielded a yellowish-brown solution.

Prior to surface modification, bare glassy carbon electrode (GCE) was sequentially polished with alumina powder (1.0, 0.3, and 0.05 μm). The electrodes were thoroughly cleaned by ultrasonication in deionized (DI) water and ethanol at a 1:1 (*w*/*w*) ratio for 10 min each.

POC/ERGO-modified electrodes were fabricated via one-step electrochemical process in a mixed solution containing 0.3 mg/mL GO and 0.3 mM OC in 10 mM PBS. This process was carried out via CV using the polished GCE at a scan rate of 50 mV/s for 5 cycles in a potential range of −1.5 to 2.24 V (vs. Ag/AgCl), during which the electrochemical reduction of GO to ERGO, and the simultaneous polymerization of OC to POC occurred [26,36]. Afterward, the modified electrode was rinsed with DI water and gently dried under N_2_ gas to eliminate loosely bound species and residual impurities. Finally, electrochemical stabilization was performed in 10 mM PBS (pH 7.4) by CV from −0.8 V to 0.0 V at 50 mV/s for 30 cycles until a stable current response was achieved.

## 3. Results

### 3.1. Electrochemical Fabrication and Characterization of the POC/ERGO-GCE

**Figure 1:**
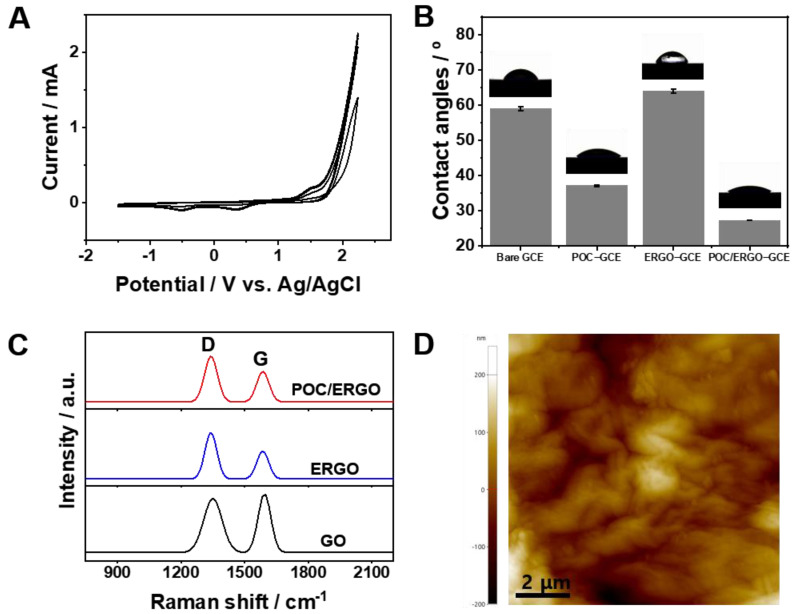
(**A**) Cyclic voltammogram recorded during the one-step electrochemical co-deposition of POC and ERGO on GCE in 10 mM PBS containing OC and GO, showing the progressive formation of the POC/ERGO composite film. (**B**) Contact angle measurements of water droplets on bare GCE, ERGO-GCE, POC-GCE, and POC/ERGO-GCE to evaluate surface wettability. (**C**) Raman Spectra of GO, ERGO-GCE, and POC/ERGO-GCE to analyze structural changes after modification. (**D**) AFM topography image of POC/ERGO-GCE.

The one-step electrochemical co-deposition process of POC/ERGO-GCE was first examined by cyclic voltammetry (Figure 1A). The cyclic voltammograms were recorded for five consecutive cycles within a potential window of −1.5 to 2.24 V vs. Ag/AgCl at a scan rate of 50 mV s^−1^ in a mixed OC/GO solution containing 0.3 mg mL^−1^ GO and 0.3 mM OC. During repeated potential cycling, the current response gradually increased with increasing cycle numbers, indicating the progressive formation of a composite film on the electrode surface. In this process, the cathodic region is associated with the electrochemical reduction of GO, while the increase in anodic current at higher potentials corresponds to the electropolymerization of OC. These results suggest that the POC/ERGO composite layer is formed through a simultaneous and integrated electrochemical process.

To further elucidate the formation mechanism, the individual electrochemical processes were separately investigated (Figure S1). As shown in Figure S1A, two cathodic peaks located at approximately −0.75 and −0.4 V are observed during the reduction of GO, which can be attributed to the stepwise removal of oxygen-containing functional groups such as epoxy, hydroxyl, and carbonyl [39]. The decrease in cathodic peak intensity with successive cycles indicates the progressive reduction of GO to ERGO. In contrast, the electropolymerization of OC exhibits a different electrochemical behavior (Figure S1B). Rather than a well-defined redox peak, a continuous increase in anodic current is observed at higher potentials (~1.5–2.24 V) during repeated potential cycling. This behavior is attributed to the electro-oxidation of phenolic groups in OC, leading to the formation of reactive intermediates that subsequently undergo coupling reactions to generate a polymeric POC film on the electrode surface [26,34,40]. The gradual increase in current with successive cycles indicates the progressive growth of the polymer layer, which is characteristic of electropolymerization processes of phenolic compounds [41]. Furthermore, repeated potential cycling was performed as an activation step to stabilize the electrode surface (Figure S1C). The cyclic voltammograms were recorded for 30 cycles in 10 mM PBS (pH 7.4) at a scan rate of 50 mV s^−1^. During this process, the current response gradually stabilized, indicating the formation of a stable electroactive interface. Such electrochemical activation is known to improve the reproducibility and stability of modified electrodes by removing loosely bound species and promoting the reorganization of the surface structure [26,34]. The surface characteristics of the fabricated electrodes were then evaluated by contact angle measurements and Raman spectroscopy (Figure 1B,C). The wettability of the electrode surface is known to be strongly influenced by surface chemistry and functional groups. The bare GCE exhibited a contact angle of 58.90 ± 0.58°, while the ERGO-GCE showed value of 63.89 ± 0.54°, reflecting partial restoration of the hydrophobic graphitic structure after GO reduction. The POC-GCE displayed a lower contact angle of 37.06 ± 0.15°, which can be attributed to the presence of hydrophilic phenolic functional groups. Notably, the POC/ERGO-GCE showed the lowest contact angle of 27.27 ± 0.11°, indicating significantly enhanced surface hydrophilicity. This improvement in wettability facilitates electrolyte penetration and promotes analyte diffusion at the electrode/electrolyte interface.

Raman spectroscopy was employed to investigate the structural evolution of graphene-based materials (Figure 1C). The GO typically presents characteristic D and G bands at approximately ~1350 cm^−1^ and ~1580 cm^−1^, respectively. Upon electrochemical reduction, the ERGO showed an increase in the intensity ratio of the D and G bands (I_D_/I_G_) from 0.9295 (GO) to 1.6683 (ERGO), indicating the removal of oxygen-containing groups and the formation of smaller sp^2^ domains with increased defect density [36,42]. In this case of the POC/ERGO composite, the Raman spectrum demonstrated a similar D/G band profile with an I_D_/I_G_ ratio of 1.5011, suggesting that the graphene structure was successfully reduced and retained after the co-deposition process. No distinct additional peaks corresponding to POC were observed, which can be attributed to the relatively low Raman activity of the polymer layer [43,44].

AFM characterization of the POC/ERGO coating was performed to investigate its surface morphology (Figure 1D). The AFM image revealed a continuous and homogeneous surface without obvious cracks or large defects. The POC/ERGO also exhibited a wrinkled and sheet-like morphology characteristic of graphene-based materials, consistent with the surface structure commonly observed for electrochemically reduced graphene oxide (ERGO) films [45]. In addition, the coating exhibited a relatively rough morphology, with an average roughness (S_a_) of 57.1 nm and a root-mean-square roughness (S_q_) of 77.8 nm. AFM height analysis yielded a maximum peak height (S_p_) of 295.7 nm, a maximum valley depth (S_v_) of 327.5 nm, and a maximum surface height (S_z_) of 623.2 nm, indicating pronounced vertical surface features across the deposited coating. These parameters collectively demonstrate the presence of nanoscale surface features and height variations over the modified electrode surface. Such textured morphology may provide increased interfacial contact area and favorable sites for analyte interaction, further supporting the successful formation of the POC layer with ERGO framework during the one-pot electropolymerization process.

**Figure 2:**
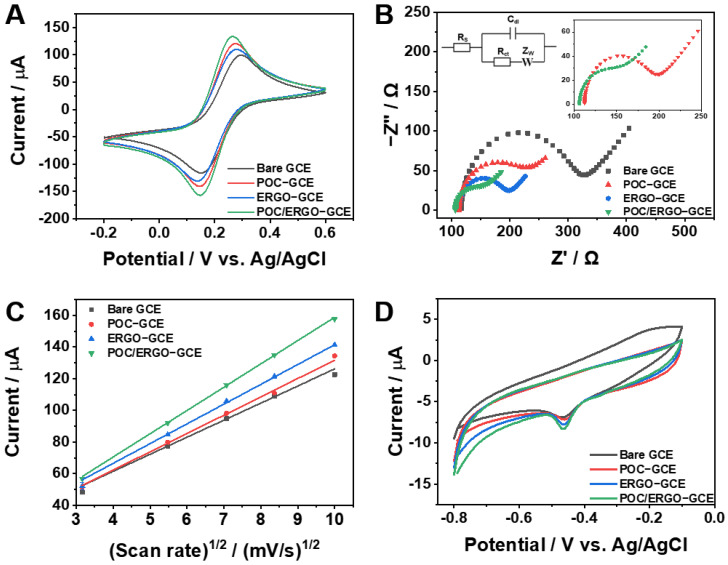
(**A**) Cyclic voltammograms of bare GCE, POC-GCE, ERGO-GCE, POC/ERGO-GCE recorded in 0.1 M KNO_3_ containing 10 mM [Fe(CN)_6_]^3−^ at scan rate of 100 mV s^−1^, (**B**) Nyquist plots of the corresponding electrodes measured in 0.1 M KNO_3_ containing 10 mM [Fe(CN)_6_]^3−^ (inset: enlarged view of POC-GCE, and ERGO-GCE) (**C**) Plots of anodic peak current (I_pa_) vs. the square root of scan rate for the determination of electrochemically active surface area. (**D**) Cyclic voltammograms of bare GCE, POC-GCE, ERGO-GCE, and POC/ERGO-GCE in 10 mM PBS containing 500 μM NFT at a scan rate of 50 mV s^−1^.

The electrochemical characteristics of the fabricated electrodes were systematically investigated to evaluate their interfacial properties and electron transfer behavior. Figure 2A presents the cyclic voltammetric response of bare GCE, POC-GCE, ERGO-GCE, and POC/ERGO-GCE in the presence of the redox probe [Fe(CN)_6_]^3−^. All electrodes exhibited well-defined redox peaks, indicating reversible electrochemical behavior. Compared to bare GCE, ERGO-GCE showed enhanced peak currents and a reduced peak-to-peak separation (ΔE_p_), which can be attributed to the increased electroactive surface area and improved electron transfer capability of the conductive ERGO layer. Similarly, POC-GCE exhibited a moderate increase in peak current, suggesting the successful formation of the polymer layer. Notably, POC/ERGO-GCE displayed the highest peak current and the smallest ΔE_p_ among all electrodes, indicating a synergistic effect between the conductive ERGO framework and the polymeric POC layer, which facilitates electron transfer at the electrode interface. Electrochemical impedance spectroscopy (EIS) analysis further confirmed the interfacial charge transfer properties of the modified electrodes (Figure 2B). As shown in the Nyquist plots, bare GCE exhibited the largest semicircle diameter, corresponding to the highest charge transfer resistance (R_ct_). In contrast, ERGO-GCE showed a significantly reduced semicircle, indicating enhanced electron transfer kinetics at the electrode interface. The POC-GCE showed a slightly increased R_ct_ compared to ERGO-GCE, which can be attributed to the partial blocking effect of the polymer layer. Importantly, the POC/ERGO-GCE exhibited the smallest semicircle diameter among the tested electrodes, demonstrating the lowest R_ct_ and confirming the enhanced interfacial electron transfer resulting from the synergistic integration of POC and ERGO.

To further evaluate the electrochemically active surface area (ECSA), the anodic peak current (I_pa_) was plotted against the square root of the scan rate (*v*^1/2^) (Figure 2C). A linear relationship was observed for all electrodes, indicating a diffusion-controlled process. The calculated ECSA values were 0.0472 cm^2^ (bare GCE), 0.0506 cm^2^ (POC-GCE), 0.0547 cm^2^ (ERGO-GCE), and 0.0644 cm^2^ (POC/ERGO-GCE), respectively. The POC/ERGO-GCE exhibited the largest ECSA, which can be attributed to the combined effects of the high surface area of ERGO and the porous polymeric structure of POC, providing more active sites for electrochemical reaction.

The electrochemical response of the modified electrodes toward NFT was further evaluated by cyclic voltammetry (Figure 2D). The POC/ERGO-GCE exhibited the highest reduction current compared to the other electrodes, indicating an enhanced electrochemical response toward NFT. This improved performance can be attributed to the combined effects of increased electroactive surface area, improved interfacial electron transfer, and favorable surface properties of the POC/ERGO composite. In addition, the electropolymerized POC layer may provide favorable interaction sites for NFT through π-π and hydrogen bonding related interactions [46], promoting local analyte accumulation at the electrode interface. The synergistic combination of enhanced charge transfer and analyte accumulation is therefore considered responsible for the improved sensing response of the POC/ERGO-GCE.

**Figure 3:**
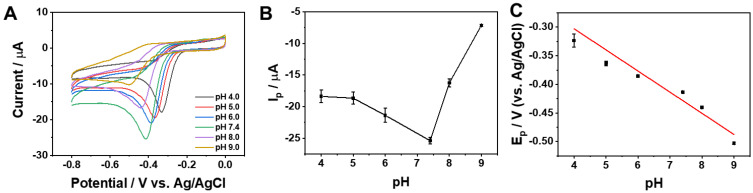
Effect of solution pH on the electrochemical response of NFT at the POC/ERGO-GCE. (**A**) Cyclic voltammograms recorded in 10 mM PBS solutions with pH values range from 4.0, 5.0, 6.0, 7.4, 8.0, and 9.0 in the presence of 500 μM of NFT. (**B**) Variation of the cathodic peak current (I_pc_) as a function of pH. (**C**) Linear relationship between the cathodic peak potential (E_pc_) and pH.

The influence of solution pH on the electrochemical response of NFT at the POC/ERGO-GCE was investigated by CV in the pH range of 4 to 9 (Figure 3A). As shown in Figure 3B, the reduction peak current increased with increasing pH from 4 to 7.4 and subsequently decreased to a higher pH value. The maximum cathodic current was observed at pH 7.4, indicating that this condition provides the most favorable environmental for NFT reduction. The reduction peak potential shifted toward more negative values with increasing pH (Figure 3C), following the linear relationship E_p_ (V) = −0.03697 pH − 0.1549 (R^2^ = 0.91). This pH-dependent potential shift suggests the involvement of protons in the electrochemical reduction process of NFT. Based on these results, pH 7.4 was selected for subsequent electrochemical measurements.

### 3.2. Electrochemical Detection of Nitrofurantoin

**Figure 4:**
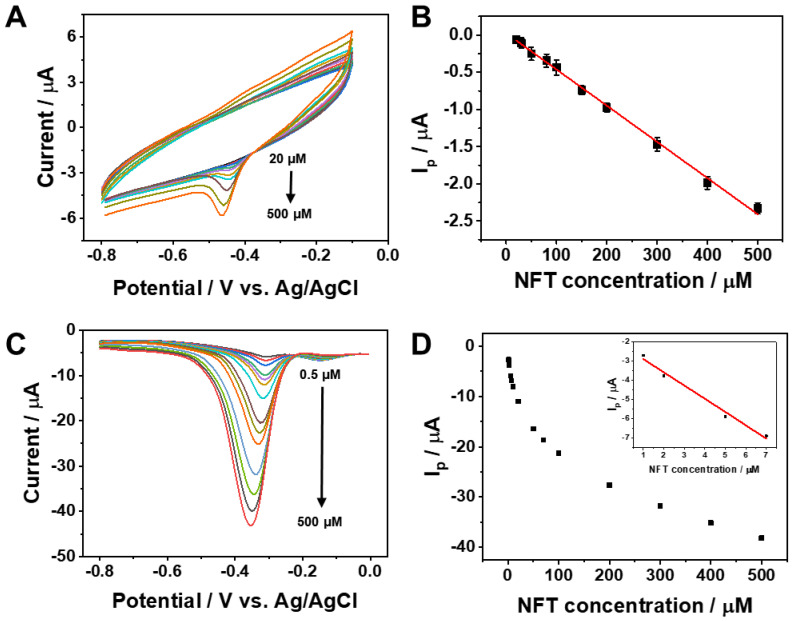
(**A**) CV of the POC/ERGO-GCE recorded in 10 mM PBS containing different concentrations of NFT (20–500 μM) at a scan rate of 50 mV s^−1^. (**B**) Corresponding calibration plot of the reduction peak current vs. NFT concentration derived from the (**A**) (S/N = 3). (**C**) DPV responses of the POC/ERGO-GCE in 10 mM PBS with various concentrations of NFT (0.5–500 μM). (**D**) Calibration plot of the reduction peak current vs. NFT concentration (1–500 μM) obtained from the DPV measurements (S/N = 3).

The electrochemical detection performance of the POC/ERGO-GCE toward NFT was evaluated using CV and differential pulse voltammetry (DPV) in Figure 4. Figure 4A shows the CV response of the POC/ERGO-GCE in 10 mM PBS (pH 7.4) containing different concentrations of NFT (20 to 500 μM). As the concentration of NFT increased, the cathodic peak current gradually increased, indicating a concentration-dependent electrochemical response. This behavior suggests that the reduction of NFT at the electrode surface is effectively monitored by the POC/ERGO-modified interface. The corresponding calibration plot (Figure 4B) shows a linear relationship between the reduction peak current and NFT concentration over the range of 20–500 μM, which can be expressed as I_p_ (μA) = −0.00486 *C*_NFT_ (μM) − 2.3028 with a correlation coefficient (R^2^) of 0.9975. The limit of detection (LOD), calculated based on a 3σ/*S*, where σ is the standard deviation of the lowest concentration signal and S is the slope of the calibration curve, was determined to be 19.59 μM.

To further improve the detection sensitivity, DPV measurements were performed (Figure 4C). The POC/ERGO-GCE showed well-defined reduction peaks with increasing NFT concentration in the range of 0.5–500 μM, demonstrating a broader detection range compared to CV. Although a measurable reduction peak was observed at 0.5 μM, this concentration was excluded from the linear regression analysis because it deviated from the linear trend observed at higher concentrations. The corresponding calibration plot (Figure 4D) shows a linear relationship between peak current and concentration of NFT, which can be expressed as I_p_ (μA) = −0.68507 *C*_NFT_ (μM) − 2.2214 with an R^2^ value of 0.9867. The LOD was calculated based on a 3σ/*S*, was determined to be 78.90 nM.

Compared with CV, DPV provided a more sensitive response due to its higher signal resolution and reduced capacitive current contribution. These results indicate that the POC/ERGO-GCE enables reliable and sensitive electrochemical detection of NFT over a wide concentration range.

To further evaluate the analytical performance of the proposed sensor, the results were compared with previously reported electrochemical and fluorescence methods for NFT detection, as summarized in Table 1 and Table S1. Although some previously reported sensors exhibited lower LOD values, the proposed POC/ERGO-GCE demonstrated competitive analytical performance with a broad linear detection range and practical advantages associated with its fabrication strategy. In particular, the POC/ERGO sensing interface was constructed through a simple one-pot electrochemical co-deposition process without requiring multi-step material synthesis, additional coating procedures, or post-treatment steps, supporting a straightforward and binder-free fabrication approach for NFT sensing. POC/ERGO-GCE exhibits comparable or improved detection performance in terms of linear range and LOD, demonstrating its effectiveness for NFT sensing.

### 3.3. Selectivity, Reproducibility, Stability, and Real Sample Analysis

**Figure 5:**
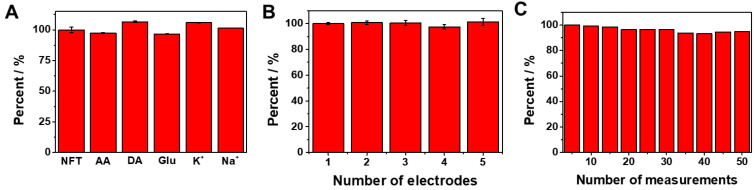
(**A**) Relative current responses of the POC/ERGO-GCE toward 100 μM of NFT and potential interfering species (1 mM of AA, DA, Glucose, K^+^, Na^+^) (*n* = 3). (**B**) Reproducibility of the POC/ERGO-GCE toward 100 μM of NFT (*n* = 3). (**C**) Operational stability of the POC/ERGO-GCE evaluated over 50 consecutive measurements toward 200 μM of NFT.

The selectivity of the POC/ERGO-GCE toward NFT was evaluated in the presence of potential interfering species, including ascorbic acid (AA), dopamine (DA), glucose, K^+^, and Na^+^ (Figure 5A). The electrochemical measurements were performed using 100 μM NFT and 1 mM of each interfering species, corresponding to a ten-fold higher concentration than that of NFT.

As shown in Figure 5A, the POC/ERGO-GCE showed a significantly higher current response toward NFT compared to the interfering species under identical conditions. The signals obtained from AA, DA, glucose, K^+^, Na^+^ were negligible relative to that of NFT, indicating minimal interference even at elevated concentrations. These results demonstrate that the proposed sensor enables reliable detection of NFT without significant signal distortion from commonly coexisting species. Selectivity measurements were performed in triplicate, and the relative standard deviation (RSDs) were found to be within 1.48% (*n* = 3), confirming the good repeatability of the selectivity test.

The reproducibility of the POC/ERGO-GCE was further evaluated using five independently fabricated electrodes toward 100 μM of NFT (Figure 5B). The five independently fabricated POC/ERGO-GCE electrodes were prepared under identical fabrication conditions. The relative current responses showed only minor variation among the electrodes, demonstrating good fabrication reproducibility of the proposed sensing platform.

The operational stability of the POC/ERGO-GCE was evaluated by performing 50 consecutive measurements in the presence of 200 μM of NFT. As shown in Figure 5C, the sensor retained 90.37% of its initial current response after repeated measurements, indicating acceptable operational stability of the fabricated sensing interface during continuous electrochemical analysis.

Human serum was selected as a representative complex biological matrix to evaluate the practical applicability of the proposed sensor. The determination of NFT in human serum samples was performed using the standard addition method. The human serum samples were diluted 10-fold with PBS (pH 7.4) prior to analysis and spiked with known concentrations of NFT. As shown in Figure S2, the blank serum sample exhibited no observable NFT reduction peak under the applied experimental conditions. Upon spiking with increasing concentration of NFT (10–200 μM), the reduction peak current increased progressively, demonstrating a concentration-dependent response in the human serum matrix. The recoveries were found to be in the range from 86.27% to 99.47% with RSD values between 0.15% and 3.24% (Table 2). Although slightly lower recoveries were observed at the lowest spiking levels, likely due to matrix effects associated with the complex serum environment, the overall recovery and precision results demonstrate satisfactory accuracy and reproducibility of the POC/ERGO-GCE for NFT detection in complex biological matrices. These results support its potential for practical bioanalytical applications.

## 4. Conclusions

In this study, a POC/ERGO nanocomposite was successfully developed through a one-step electrochemical co-deposition strategy and applied for the electrochemical detection of NFT. The improved sensing performance can be attributed to the increased electrochemically active surface area and the favorable electron transfer behavior of the POC/ERGO-GCE. DPV measurements demonstrated a wide linear detection range, low detection limit, and excellent anti-interference capability, confirming the reliability of the proposed sensing platform. Furthermore, the sensor exhibited good reproducibility and selectivity toward NFT. These results suggest that the POC/ERGO-GCE provides a promising foundation for electrochemical sensing of antibiotic compounds. Further studies will focus on extending the applicability of this system to complex sample environments.

## Figures and Tables

**Table 1:** Comparison of previously reported electrochemical methods for nitrofurantoin detection.

Sensing Material	Detection Method	Linear Range (μM)	LOD (μM)	Fabrication Strategy & Preparation Process
CeO_2_ NPs [47]	DPV/CV	20–100	7.81	Hydrothermal synthesis of CeO_2_ nanoparticles followed by electrode modification
Ag-Pd NCs [48]	DPV	5–210	3.2	Chemical synthesis of Ag-Pd nanocubes followed by electrode coating
lanthanum molybdate nanospheres [49]	DPV	0.01–144	0.072	Co-precipitation synthesis followed by drop-casting onto the electrode surface
Ag-Ni(OH)_2_ NPs [50]	CV/DPV	0.11–13, 13–212	0.079	Chemical precipitation of Ni(OH)_2_ and Ag-Ni(OH)_2_ nanoparticle formation followed by electrode coating
Fe/Graphene/Porphyrin [51]	DPV	0.5–200	0.246	Solution preparation of Fe/graphene/porphyrin composite followed by surface modification
Ru/NiFe-LDH-Mxene [3]	LSV	0.01–275	0.0022	Hybrid material synthesis followed by electrochemical deposition
Bi_2_S_3_–TiO_2_/HNTs [52]	LSV	0–275	0.0032	Composite synthesis followed by drop-casting and drying
POC/ERGO [This work]	DPV/CV	1–500	0.0789	One-pot electrochemical co-deposition of OC and GO with simultaneous electropolymerization and GO reduction (binder-free)

**Table 2:** Determination of NFT in human serum samples using the POC/ERGO-GCE.

Added (μM)	Found (μM)	RSD (%)	Recovery (%)
10	8.63	3.24	86.27
20	17.6	0.93	87.97
50	49.7	0.28	99.47
100	93.3	2.33	93.29
200	190.8	0.15	95.40

## Data Availability

The data presented in this study is available on request from the corresponding author.

## Supplementary Materials

The following supporting information can be downloaded at: https://www.mdpi.com/article/10.3390/s26123682/s1, Figure S1: (A) Cyclic voltammograms (5 cycles) for the electrochemical reduction of GO to ERGO in 10 mM PBS containing 0.3 mg/mL GO at a scan rate of 50 mV s^−1^. (B) Cyclic voltammograms (5 cycles) recorded during the electropolymerization of OC to POC in 10 mM PBS containing 0.3 mM OC at a scan rate of 50 mV s^−1^. (C) Cyclic voltammograms (30 cycles) recorded for electrochemical activation of the POC/ERGO-GCE in 10 mM PBS at a scan rate of 50 mV s^−1^; Figure S2. Real sample analysis of NFT in human serum. DPV responses obtained from blank and spiked with different NFT concentration (10–200 μM); Table S1: Comparison of other methods for nitrofurantoin detection. Refs. [53,54,55,56] are cited in the Supplementary Materials.
