# Supplementary material for: One-Step Electrochemical Fabrication of Poly O-cresolphthalein Complexone and Electrochemically Reduced Graphene Oxide Modified Electrode for Detection of Nitrofurantoin
Source: Sensors (Basel). 2026 Jun 9;26(12):3682. doi: 10.3390/s26123682 (PMC13307319; doi:10.3390/s26123682)
Supplement: Supplementary file 1 [file sensors-26-03682-s001.zip › sensors-4335262-supplementary.pdf]

Supplementary material

for

One-Step Electrochemical Fabrication of Poly O-cresolphthalein complexone and Electrochemically Reduced Graphene Oxide Modified Electrode for Detection of Nitrofurantoin

Ju Sung Kim †, Da Eun Oh †, and Tae Hyun Kim *

Department of Chemistry, Soonchunhyang University, Republic of Korea

* Correspondence to. thkim@sch.ac.kr (T. H. Kim); Tel.: +82-41-530-4722

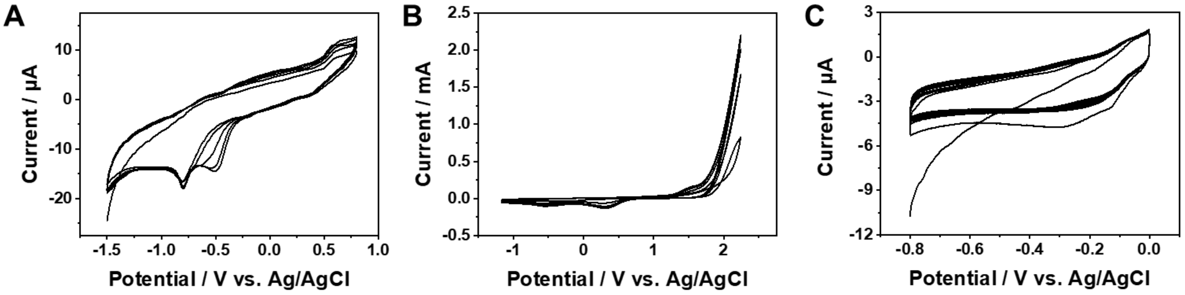

Figure S1. (A) Cyclic voltammograms (5 cycles) for the electrochemical reduction of GO to ERGO in 10 mM PBS containing 0.3 mg/mL GO at a scan rate of 50 mV s^{-1}. (B) Cyclic voltammograms (5 cycles) recorded during the electropolymerization of OC to POC in 10 mM PBS containing 0.3 mM OC at a scan rate of 50 mV s^{-1}. (C) Cyclic voltammograms (30 cycles) recorded for electrochemical activation of the POC/ERGO-GCE in 10 mM PBS at a scan rate of 50 mV s^{-1}.

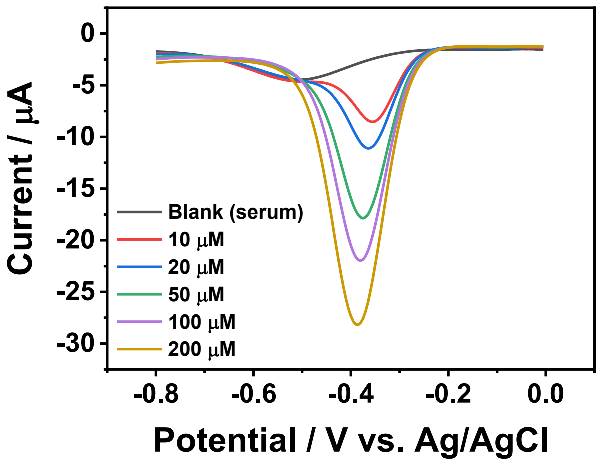

Figure S2. Real sample analysis of NFT in human serum. DPV responses obtained from blank and spiked with different NFT concentration (10-200 µM).

Table S1. Comparison of other methods for nitrofurantoin detection.

Sensing material	Detection method	Linear range (μM)	LOD (μM)	REF
Ag-NPs	Fluorescence	10-60	0.98	[53]
Cu NCs	Fluorescence	5-120	0.73	[54]
Zn2+@Eu-bpdc	Fluorescence	-	0.079	[55]
2D-MOF	Colorimetry	-	0.091	[56]
POC/ERGO	Electrochemistry	1 - 500	0.0789	This work
